# Reticular Dysgenesis and Mitochondriopathy Induced by Adenylate Kinase 2 Deficiency with Atypical Presentation

**DOI:** 10.1038/s41598-019-51922-2

**Published:** 2019-10-31

**Authors:** Lina Ghaloul-Gonzalez, Al-Walid Mohsen, Anuradha Karunanidhi, Bianca Seminotti, Hey Chong, Suneeta Madan-Khetarpal, Jessica Sebastian, Catherine Walsh Vockley, Miguel Reyes-Múgica, Mark T. Vander Lugt, Jerry Vockley

**Affiliations:** 1 Division of Medical Genetics, Department of Pediatrics, University of Pittsburgh, Pittsburgh, PA USA; 2 Department of Human Genetics, Graduate School of Public Health, University of Pittsburgh, Pittsburgh, PA USA; 3 Division of Pulmonology, Allergy and Immunology, Department of Pediatrics, University of Pittsburgh, Pittsburgh, PA USA; 4 Division of Pediatric Pathology, Department of Pathology, University of Pittsburgh, Pittsburgh, PA USA; 5 Division of Blood and Marrow Transplantation and Cellular Therapies, Department of Pediatrics, University of Pittsburgh, Pittsburgh, PA USA

**Keywords:** Disease genetics, Genetics research

## Abstract

Reticular dysgenesis is an autosomal recessive form of severe combined immunodeficiency (SCID) that usually manifests in newborns. It is a unique example of an immune deficiency that is linked to dysfunctional mitochondrial energy metabolism and caused by adenylate kinase 2 **(**AK2) deficiency. It is characterized by an early differentiation arrest in the myeloid lineage, impaired lymphoid maturation, and sensorineural hearing loss. In this study, a novel *AK2* homozygous mutation, c.622 T > C [p.Ser208Pro], was identified in an Old Order Amish patient through whole exome sequencing. Functional studies showed that the patient’s cells have no detectable AK2 protein, as well as low oxygen consumption rate (OCR), extracellular acidification rate (ECAR) and proton production rate (PPR). An increased production of reactive oxygen species, mitochondrial membrane permeability, and mitochondrial mass, and decreased ATP production, were also observed. The results confirm the pathogenicity of the *AK2* mutation and demonstrate that reticular dysgenesis should be considered in Amish individuals presenting with immune deficiency. We also describe other pathophysiological aspects of AK2 deficiency not previously reported.

## Introduction

Reticular dysgenesis (RD) (MIM267500; aleukocytosis) is an autosomal recessive form of severe combined immunodeficiency (SCID) resulting from defects in the adenylate kinase 2, *AK2*, gene located on chromosome 1p35.1. Mitochondrial dysfuntion appears to be unique to AK2 deficiency among the immune deficiencies^1–3^. RD is one of the most rare forms of SCID and also one of the most severe due to lack of not only lymphocytes but also granulocytes, often leading to fatal neonatal sepsis^1,4^. Additionally, individuals with RD have hypoplasia of the thymus and secondary lymphoid organs^1,4^. Current treatment of RD is limited to hematopoietic stem cell transplantation (HSCT)^4,5^.

AK2 is a phosphotransferase enzyme localized in the mitochondrial intermembrane space and catalyzes the reversible transfer of a phosphoryl group from ATP to AMP giving 2 ADP molecules. ADP is then transported into the mitochondrial matrix by the ADP-ATP carriers that, in exchange, export ATP synthesized by oxidative phosphorylation (OXPHOS) into the cytosol. Balanaced ADP production and transport is critical to cellular energy homeostais since OXPHOS activity is dependent on matrix ADP levels^1,2^.

While nine adenylate kinase isoforms have been identified in various tissues, AK2 is the only isoenzyme identified in bone marrow white blood cells pregenitors, leading to the immune deficiency in AK2 deficient patients^1,2,6^. AK2 is also expressed in the inner ear and its dysfunction is likely the cause of the hearing defects observed in patients with AK2 deficiency^1^.

In this study, an AK2 deficiency identified in a five-year old Amish male with a history of immunodeficiency is described as well as the cellular pathophysiology induced by this defect.

## Material and Methods

### Study design

This study focused on the Amish population for discovery of novel genetic disorders through whole exome sequencing. The current patient was recruited into this research study after extensive genetic testing failed to diagnose known causes of combined immune deficiency.

### Consent

Informed consent was obtained and approved for all participants in accordance to the University of Pittsburgh IRB approved protocol #PRO11070174 for clinical genomic studies. For underaged participants, parental informed consent was obtained and approved. All methods were performed in accordance with the relevant guidelines and regulations outlined by the IRB. Whole exome sequencing on a research basis was offered to the family who agreed to participate in the study.

### Case selection

This case was selected as part of a study to diagnose novel genetic disorders in the Amish population in western Pennsylvania. The patient was recruited into this study because of his combined immune deficiency of unknown etiology.

### Case description

**Figure 1:**
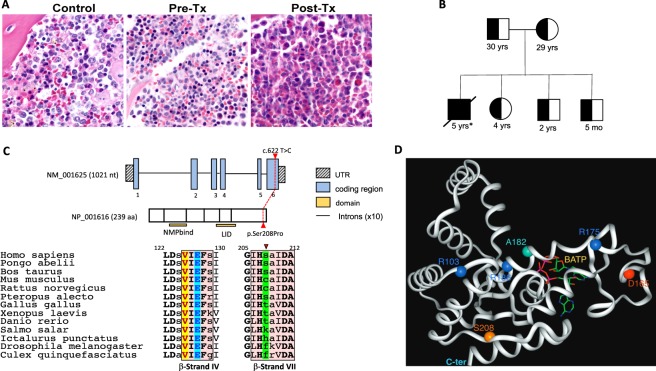
H&E staining of the bone marrow, Pedigree, AK2 gene and protein structure. (**A**) H&E staining of the bone marrow. Control; bone marrow from an age-matched individual showing adequate cellularity with all normal hemopoietic cell lines represented and without predominance of any particular lineage. Pre-Tx; pre-transplant bone marrow biopsy from the patient at 13 months of age before bone marrow transplant showing myeloid maturation only through the promyelocyte/myelocyte stage. Only occasional neutrophils were seen. Post-Tx; post-transplant bone marrow biopsy from affected patient after bone marrow transplant showing normocellular bone marrow with trilineage hematopoiesis (all images at 100X). (**B**) Pedigree of the family identified with mutations in the *AK2* gene. Both parents and siblings are unaffected and heterozygous for the mutation while the patient is homozygous. Ages are representative of the individual ages at the time of manuscript submission. Asterisk denotes the age of the patient when he died following bone marrow transplant complication. (**C**) Structure of the *AK2* gene (GenBank: NM_001625), location of the Ser208Pro mutation relative to the polypeptide stretch, and homology alignment of the AK2 β-strands IV (L125-I129) and VII (G206-A212) regions. The secondary structure assignments are according to the human AK2 crystal structure atomic coordinates, PDB 2C9Y, highlighted in pink boxes. Residues in bold capital letters are invariants in all species examined, only 13 are shown. Residues in capital letters (not bold) are highly conserved, and residues in small letters seem dispensable. Highlighted in green is the Ser208 position. (**D**) Ribbon representation of the AK2 protein 3D structure, PDB 2C9Y, with the position of Ser208 depicted (replaced with a Pro in the patient of this study), plus the position of other previously reported mutations in the AK2 protein. BATP (letters in yellow) is the ATP binding domain.

A five-year old Old Order Amish male first presented at ten months of age with *Haemophilus influenza* and *Pseudomonas aeruginosa* sepsis and pneumonia. At his first presentation, he had neutropenia (ANC 1030 cells/µL), T and B cell lymphopenia (143 cell/µL and 15 cells/µL, respectively), and hypogammaglobulinemia (IgG of 211 mg/dL). Proliferation to phytohemagglutinin (PHA) was decreased (25.2%; normal >49.9%). Combo-Chip Array studies identified a 16p11.2 duplication as well as regions of homozygosity on chromosomes 1, 2, and 10. Testing for immunodeficiency syndromes in the Amish related to known founder mutations were normal. A bone marrow aspirate and biopsy at 13 months of age showed maturation arrest, which occurred primarily through the promyelocyte/myelocyte stage, showing only an occasional neutrophil (Fig. 1A), and the patient was started on G-CSF for neutropenia with adequate response. He developed bronchiectasis due to recurrent pulmonary infections and at 3 years of age, he developed refractory primary CMV viremia. Because of this, he underwent a hematopoietic stem cell transplantation (HSCT) from a mismatched related donor (maternal) for combined immunodeficiency. He engrafted with full donor chimerism; however, he developed neutropenia and complete recipient chimerism in the myeloid lineage by six months post-transplant with continued complete donor chimerism in CD3^+^ cells. He was diagnosed with moderate to severe sensorineural hearing loss at 4 years of age with absent otoacostic emissions (OAEs) following an evaluation for abnormal speech. He had a history of right failed newborn hearing screen. He underwent a second HSCT from the same donor 2 years later, which was complicated by engraftment syndrome and severe veno-occlusive disease of the liver, which was ultimately fatal (see Supplementary Materials for complete clinical synopsis).

### Whole exome sequencing

DNA was extracted from blood samples from all six subjects (pedigree, Fig. 1B). Whole exome sequencing was performed on DNA samples from unaffected parents and the affected patient by BGI Americas Corporation. Sequencing via the Illumina Hiseq. 2000 was performed with library construction using Agilent SureSelect Human All Exon V4 (51 Mb) with a target of 100X coverage per sample. FASTQ files were delivered to us for analysis.

### Sequence analysis

Fastq files from both parents and the affected child were imported into CLC genomics workbench 9.5.1 program (CLC bio QIAGEN, Germany, http://www.clcbio.com/products/clc-genomics-workbench/). Reads were aligned to homo-sapiens reference sequence hg19 to create VCF files. VCF files were then imported into the Omicia Opal platform (Omicia Inc, CA, https://www.omicia.com) for interpretation. The three individuals had an average variant number between 138,000 and 139,500. Since autosomal recessive disorders due to founder mutations are the most common mode of inheritance for the genetic disorders in the Amish, the following criteria were used for analysis: (1) homozygous variants in the patient that were heterozygous in the parents; (2) minor allele frequency (MAF) cutoff of <1% in 1000 genomes, NHLBI-ESP 6500 databases (http://evs.gs.washington.edu/EVS/) and Exome Aggregation Consortium (ExAC) Server (Cambridge, MA [09/2015]) databases (http://exac.broadinstitute.org); (3) coding changes in the protein. Using this filtering pipeline, we were able to decrease the number of variants to 26. Analysis was limited to only to the part of the exome relevant to the patient phenotype. The human phenotype ontology (HPO) term for immune deficiency was used to further refine assignment of causation. Sanger sequencing was performed on all family members to confirm mutation status for the *AK2* gene.

### Western blot analysis

Western blotting of total cell lysates was performed as previously described^7^. Primary rabbit anti Ak2 monoclonal antibody (AB157206, Abcam, Cambridge, MA) or primary mouse anti β-Actin monoclonal antibody (A1978, Sigma-Aldrich, St. Louis, MO) were used followed by HRP-conjugated secondary antibody.

### Immunofluorescence staining of fibroblasts and bone marrow

Patient (PT) fibroblasts were obtained using standard culture protocol of a skin biopsy obtained from the patient at the UPMC Children’s Hospital of Pittsburgh, PA. Normal adult primary human dermal fibroblasts (HDFa), ATCC PCS201012, were obtained from ATCC (https://www.atcc.org), Manassas, VA, and used as Control (CT). The fibroblasts were seeded at a concentration of 5Χ10^4^ cells/ml on tissue culture-treated glass cover slips and allowed to grow overnight at 37 °C in a 5% CO_2_, 95% humidity incubator. Cells were then fixed and immunostained as previously described^8^. A double primary antibody was used for the first incubation: (1) rabbit polyclonal anti-AK2 antibody (Abcam, Cambridge, MA) localized in the inter-mitochondrial membrane space, and (2) mouse monoclonal anti cytochrome c oxidase subunit 4 antibody (anti-MTCO1) localized in the inner mitochondrial membrane (Abcam, Cambridge, MA). Labeled secondary antibodies were donkey anti-rabbit secondary antibody Alexa Fluor 488 (ThermoFisher Scientific, Waltham, MA) for AK2, and donkey anti-mouse secondary antibody Alexa Fluor 555 (ThermoFisher Scientific, Waltham, MA) MTCO1. All primary antibodies cross-react with the human protein. Nuclei were counterstained with DAPI. The cover slips were then mounted using mounting media before imaging. All the images were taken using an Olympus Confocal FluoView FV1000 microscope at a magnification of 60Χ. Formalin-fixed, paraffin-embedded bone marrow tissue (FFPE) slides of bone marrow were provided by the Department of Pathology, Children’s Hospital of Pittsburgh and stained as above.

### Reverse transcription PCR

RT-PCR was used to assess AK2 mRNA expression in PT fibroblasts. CT and PT cells were grown to confluence in a T25 culture flasks. RNA extraction was performed on the cell pellets using Quick-RNA Microprep kit according to the manufacturer’s protocol (ZymorResearch, Irvine, CA). Reverse Transcription **(**RT) was performed using the random hexamer supplied with the SuperScript III First -Strand Synthesis System according to the manufacturer’s protocol (ThermoFisher Scientific, Waltham, MA). PCR was simultaneously done for GAPDH as a standard reference gene. The PCR products were then separated by 2% agarose gel electrophoresis.

### Measurement of mitochondrial respiration

Mitochondrial respiration was measured with a Seahorse XF^e^96 Extracellular Flux Analyzer (Seahorse Bioscience, Billerica, MA). Oxidative phosphorylation (OXPHOS) is measured by oxygen consumption rate (OCR) while glycolysis is measured by the generation of lactate and the consequent extracellular acidification rate (ECAR), providing real-time monitoring of mitochondrial respiration. Cells were seeded in 96-well Seahorse tissue culture microplates in growth media at a density of 80,000 cells per well. To ensure equal cell numbers, cells were seeded in cell culture plates pre-coated with Cell-Tak (BD Biosciences, San Jose, CA). All cell lines were measured with four to six wells per cell line. Then, the entire experiment was repeated. Before running the assay, cells were incubated for 1 h without CO_2_ in unbuffered DMEM. Cells were then treated with successive addition of oligomycin, carbonyl cyanide 4-(trifluoromethoxy) phenylhydrazone (FCCP), 2-deoxy-glucose (2-DG) and Rotenone/antimycin A, Seahorse XF Cell Mito Stress Test Kit, Santa Clara, CA. Initial OCR was measured to establish a baseline (basal respiration). Reserve capacity was determined after the injection of FCCP, data were reported in pmol/min for OCR. ECAR was measured and expressed as mpH/min which is a measure of the glycolytic conversion of glucose to lactate and consequently net production and extrusion of protons into the extracelluar medium. This measurement was automatically converted to proton production rate (PPR) expressed as pmol H^+^/min^9–11^.

### Measurement of superoxide production

Cell suspension containing 1 × 10^5^ cells per mL were incubated for 15 min at 37 °C with 5 µM MitoSOX Red (Invitrogen, Grand Island, NY) for superoxide production measurement. After incubation, 10,000 cells samples were analyzed in a Becton Dickinson FACSAria II flow cytometer (BD Biosciences, San Jose, CA).

### Measurement of mitochondrial mass and activity

Measurement of the mitochondrial membrane mass and membrane potential was performed in both control (CT) and patient (PT) fibroblasts. Cell suspension containing 1 × 10^5^ cells per mL were incubated for 25 min at 37 °C with 150 nM Mitotracker Green (Invitrogen, Grand Island, NY) for mitochondrial mass evaluation and with 200 nM Mitotracker Red, (Invitrogen) for mitochondrial membrane potential measurement. After incubation, 10,000 cells samples were analyzed in a Becton Dickinson FACSAria II flow cytometer (BD Biosciences).

### ATP production assay

ATP production was determined by a bioluminescence assay using an ATP determination kit (ATPlite; PerkinElmer Inc, Waltham, MA) according to the manufacturer’s instructions. The luminescence was measured in a SpectraMax i3x Platform multi-mode microplate reader system (Molecular Devices, LLC, Sunnyvale, CA). Data were reported in μmol/mg of protein.

### Statistics

Full data is presented for the subject and functional studies. Statistical significance was assessed by performing the Student’s *t*-test using GraphPad Prism version 7.00 for Windows, GraphPad Software (La Jolla, California, USA, www.graphpad.com). A p-value < 0.05 was considered significant.

## Results

### Identification of the *AK2* mutation causing the immune deficiency

Whole Exome Sequencing (WES) was performed on DNA from the patient and both unaffected parents (pedigree; Fig. 1B). Given the increased risk for homozygosity in recessive disorders in the Amish, we searched for previously undescribed, rare homozygous mutation in the patient, with MAF <1% in the ExAC (Exome Aggregation Consortium) database. A novel homozygous missense mutation c.622 T > C [p.Ser208Pro] in the last exon, exon 6, of the *AK2* gene (GenBank: NM_001625.3) was identified in the patient, for which both parents were carriers. Additionally, Sanger sequencing performed on all family members including siblings confirmed the WES findings from both parents and the affected child, while all unaffected siblings were heterozygous for the mutation (Supplementary Table 2).

### Molecular modeling and prediction of protein structural perturbations

To investigate the effect of the Ser208Pro replacement on enzyme structure and function, phylogenetic conservation and molecular modeling analyses were conducted. While phylogenetic analysis shows that the amino acid residue at the 208 position is not conserved (Fig. 1C), it also shows that Ser208 is flanked on both sides by His207, an invariant residue, and other invariant residues that are two positions away. Molecular modeling using the published recombinant human AK2 crystal structure coordinates (PDB: 2C9Y) provides insight into the critical role of the backbone atoms at position 208 in maintaining the integrity of the protein structure. The AK2 protein consists of three domains. The first is a large central CORE domain, comprising the central parallel β-sheet with several surrounding α-helices. Two small peripheral domains include a nucleoside monophosphate binding domain (NMPbind) and a LID domain, which undergo movement during catalysis^12,13^. The central CORE domain consists largely of a β-sheet comprised of β-strands I to IV and VII. Ser208 is positioned near the middle of β-strand VII, the longest and the last β-strand of the CORE β-sheet domain. Its backbone amide nitrogen and carbonyl oxygen interact with the carbonyl oxygen of Val125 and the amide nitrogen of Glu127 of the juxtaposed β-strand IV, respectively, contributing with two of the six hydrogen bonds that constitute the crucial interaction between these two parallel β-strands (Fig. 1C,D). Moreover, while the backbone amide nitrogen and carbonyl oxygen of the invariant Glu127 are hydrogen bonded with the Ser208 and Ile210, respectively, an ionic interaction between the carboxylate of Glu127 and the imidazole ring of the invariant His207 straddles the Ser208 hydrogen bonding across the two β-strands, suggesting that all of these amino acids are crucial for maintaining the integrity of the CORE β-sheet domain edge where β-strand VII is positioned. Replacement of the Ser at position 208 with a Pro is expected to introduce a kink and therefore cause misalignment of the backbone amide nitrogen and carbonyl oxygen at the 208 position along with loss of the hydrogen bond with both Val125 and Glu127 and so altering the trajectory of the β-strand VII backbone, and disrupting the Glu127 interaction with Ile210 and His207. The structural perturbations induced by a Pro, at the position 208 position would therefore be extensive and detrimental to the integrity of the β-sheet domain and is most likely to hinder proper folding. These modeling predications are consistent with the lack of protein signal reported below.

### Absence of AK2 protein in patient cells is attributable to its instability

**Figure 2:**
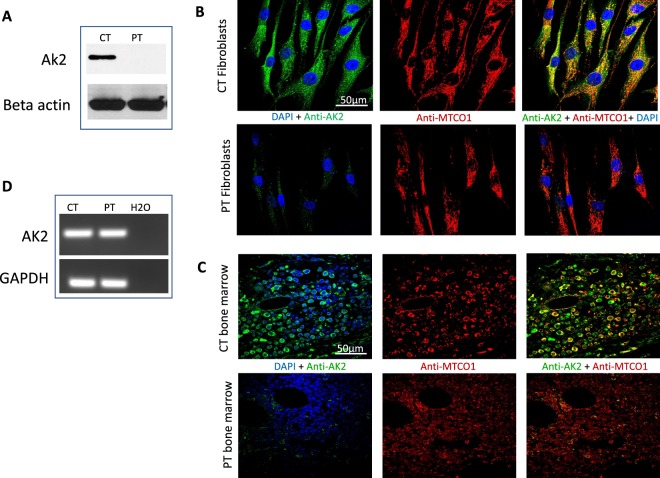
Protein and mRNA expression analysis of AK2 in cell lines derived control (CT) and patient (PT). (**A**) Fibroblast cells from CT and PT were analyzed for the presence of AK2 antigen by SDS-PAGE followed by western blotting with anti-AK2 and anti-β-actin antibodies. Twenty-five µg protein from fibroblasts’ lysates were loaded for both CT and PT (see Supplementary Figs S1 and S2 for full blot). (**B**,**C**) Immunofluorescence staining of fibroblasts and bone marrow from CT and PT. AK2 antigen was visualized with green fluorescently tagged antibodies and mitochondrial cytochrome c oxidase subunit 1 (MTCO1) was visualized with red fluorescently tagged antibodies. Nuclei were visualized with DAPI staining. The merged image shows co-localization of AK2 and MTCO1 in mitochondria as yellow/orange in the CT fibroblasts while there is essentially no green staining that would show co-localization with MTCO1 in the patient fibroblasts. Scale bar, 50 μm. (**D**) RT-PCR for AK2 and GAPDH on CT, PT and negative control (H_2_O) samples. GAPDH was used as a standard reference gene. Both CT and PT PCR reactions resulted in similar cDNA band instensity (see Supplementary Fig. S3 for full image of agarose gel electrophoresis).

To investigate the predicted structural/functional adverse effects of the *AK2* mutation, we first attempted to measure the antigen level of AK2 in patient fibroblasts and bone marrow cells. Western blot analysis failed to detect AK2 protein in patient fibroblasts compared to control, a result that was confirmed by immunofluorescene staining of patient’s pre-transplant bone marrow and fibroblasts (Fig. 2). To rule out that absence of AK2 protein signal is attributed to mRNA instability, conventional RT-PCR on control and patient fibroblasts was carried out to assess AK2 mRNA expression signal using GAPDH as standard reference gene. Both control and patient PCR reactions resulted in similar cDNA band instensity consistent with the hypothesis above that degradation occurs rapidly at the protein synthesis and/or folding level (Fig. 2).

### Characterization of mitochondrial dysfunction in AK2 deficiency in patient fibroblasts

**Figure 3:**
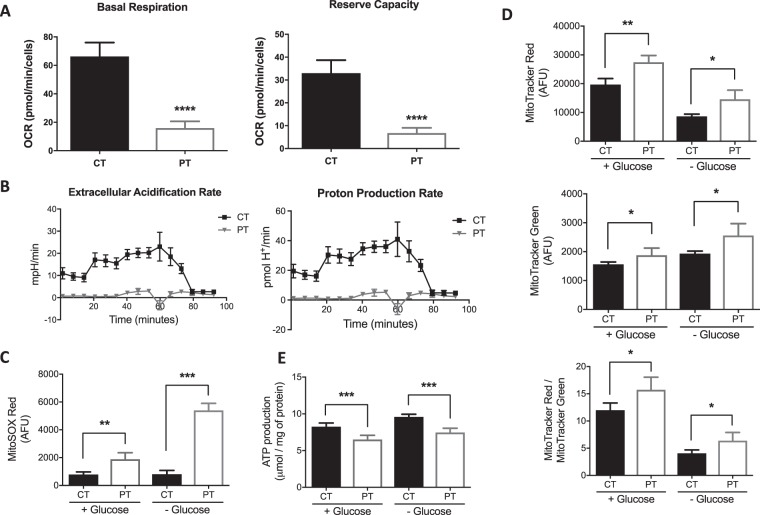
Mitochondrial studies in control (CT) and patient (PT). (**A**) Oxygen consumption rate (OCR) measured using both parameters with Seahorse XF^e^96 Extracellular Flux Analyzer: Basal respiration and reserve capacity. Both were decreased in the PT compared with CT. Data reported in pmol/min/cells. (**B**) Extracellular acidification (ECAR) and the proton production (PPR) rate using Seahorse XF^e^96 Extracellular Flux Analyzer. Both rates can be used as surrogates for lactate production attributed to anaerobic glycolysis. Both are decreased in PT compared to CT. (**C**) Reactive oxygen species (ROS) production by measuring superoxide production using MitoSOX Red with and without using glucose. ROS is higher in PT’s fibroblasts comparing with the CT, and is more pronounced when the media used is without glucose. (**D**) Measurement of the mitochondrial mass and membrane potential with MitoTracker Green and Red, respectively, revealed a significant increase in mitochondrial mass concurrent with an increase in mitochondrial membrane potential in patient fibroblasts. (**E**) ATP production assay showing significantly lower ATP in the patient cells (PT) compared to the CT.

Mitochondrial respiration in patient fibroblasts, as reflected by the whole cell oxygen consumption rate (OCR), was abnormal (Fig. 3A). This included the basal respiration rate, as well as reserve capacity, a measure of ability of the mitochondria to respond to physiological stress. Extracellular acidification rate (ECAR), an indirect analysis of the glycolytic rate and the proton production rate (PPR), was also decreased in patient cells (Fig. 3B). Additionally, superoxide production in patient fibroblasts, as measured with MitoSOX Red™, a fluorogenic dye for highly selective detection of superoxide in the mitochondria of live cells, was dramatically increased compared to control cells (Fig. 3C). Measurement of the mitochondrial mass and membrane potential with MitoTracker Green™ and Red™, respectively, revealed a significant increase in mitochondrial mass concurrent with an increase in mitochondrial membrane potential in patient fibroblasts, indicating disruption in the mitochondrial proton gradient crucial for ATP production by ATP synthase (Fig. 3D). These findings are consistent with significantly lower ATP found in patient cells (Fig. 3E).

## Discussion

This is the first report of RD attributed to an *AK2* gene mutation in the Amish population and includes cellular and molecular functional studies confirming the pathophysiologic effect of the identified mutation. Standard clinical criteria for RD include 1) absence, or very low number of T cells (CD3 T cells <300/microliter); 2) no, or very low (<10% of lower limit of normal) T cell function (as measured by response to PHA; 3) severe neutropenia (absolute neutrophil count <200/microliter) that is typically unresponsive to G-CSF; and 4) sensorineural deafness and/or absence of granulopoiesis at bone marrow examination and/or a deleterious AK2 mutation^14^. Although the *AK2* gene in this patient was in a region of homozygosity on chromosome 1, he did not meet the clinical criteria for classical RD and genetic testing for an *AK2* mutation originally was not pursued. Subsequently, WES revealed the homozygous mutation, c.622 T > C [p.Ser208Pro] in the *AK2* gene as the most likely cause of the observed immune deficiency.

AK2 is critical to the control of energy metabolism. It regulates intracellular ATP levels by catalyzing the reversible transfer of a phosphate group in the reaction ATP + AMP ↔ 2 ADP and contributes to ~60% of the ADP flux in the mitochondrial matrix^1^. Since AK2 is the only adenylate kinase expressed in the bone marrow, its absence is expected to severely disrupt mitochondrial function and oxidative phosphorylation leading to a profound block in lymphoid and myeloid cell differentiation^3^. AK2 protein is located in the inter-membrane space of mitochondria, whereas other members of the AK family are cytoplasmic (AK1, 5, 7 and 8), nuclear (AK6), or located in the mitochondrial matrix (AK3 and AK4)^3,15,16^.

While most patients with *AK2* mutations published to date had severe sepsis in the newborn period, this patient had a less severe phenotype including delayed clinical presentation of sepsis and response to G-CSF. Of note, he showed improvement in his lymphocyte production with generation of naïve T cells, unusual for patients with other causes of SCID. One case of “leaky” RD has been reported previously in an infant with known RD who developed Omenn syndrome, however, in contrast to the patient in this study, the former patient was profoundly neutropenic and had an oligoclonal T cell repertoire lacking naïve T cells^17^. Four other patients with homozygous *AK2* mutations presented with variable clinical features that include combined immunodeficiency (CID) and hypogammaglobulinemia without agranulopoiesis have also been reported^18^.

Although the patient in this study did not meet the clinical criteria for classical RD, functional studies clearly have proven pathogenicity of his mutation, with evidence of decreased mitochondrial oxygen consumption and ATP generation, increased accumulation of reactive oxygen species, and hyperpolarization of the inner mitochondrial membrane potential. Impaired oxidative phosphorylation should lead to increased glycolysis and in turn, increased lactate production. However, lactate accumulation in patient fibroblasts was normal as revealed in the ECAR and PPR experiments, presumably due to the presence of other adenylate kinase isozymes in other tissues. Lactic acidosis is not expected in patients as the limited amount produced in affected cells can quickly be metabolized by other tissues.

In summary, *AK2* joins *RAG1*, *ADA* as well as *ITCH and RMRP* genes in the list of known causes of immune deficiency in the Amish population. This reported case illustrates the importance of considering RD caused by *AK2* gene mutations in patients with immune deficiency and less severe phenotypes than “classical” RD. It also provides impetus for investigating *AK2* mutations in other patients with unexplained SCID, especially when associated with hearing loss. The demonstrated dysfunction of mitochondrial energy metabolism in patient fibroblasts suggests that treatment of the underlying mitochondriopathy might be of use while patients are waiting for a bone marrow transplant. Such treatment should not delay planned bone marrow transplantation. In addition, early recognition of RD prior to HSCT is important because, in contrast to other forms of SCID, the use of myeloablative conditioning regimens must be considered, particularly for patients receiving T cell-depleted grafts due to the increased risk of graft rejection and need to obtain myeloid engraftment to fully correct the underlying immunodeficiency^19^. This case also heighlights the importance of TREC (T cell receptor excision circles) testing as part of the newborn screening to diagnose SCID as early as possible, even if sepsis due to SCID is not present in the newborn period.

## Supplementary information

Supplementary Materials 

## Data Availability

All materials used in this study are commercially available. All data are available upon request as mandated by NIH guidelines.
