# Supplementary material for: Reticular Dysgenesis and Mitochondriopathy Induced by Adenylate Kinase 2 Deficiency with Atypical Presentation
Source: Sci Rep. 2019 Oct 31;9:15739. doi: 10.1038/s41598-019-51922-2 (PMC6823482; doi:10.1038/s41598-019-51922-2)

Reticular Dysgenesis and Mitochondriopathy Induced by Adenylate Kinase 2 Deficiency with Atypical Presentation

Lina Ghaloul-Gonzalez[1,2]*, Al-Walid Mohsen[1,2], Anuradha Karunanidhi[1], Bianca Seminotti[1], Hey Chong[3], Suneeta Madan-Khetarpal[1,2], Jessica Sebastian[1], Catherine Walsh Vockley[1], Miguel Reyes-Múgica[4], Mark T. Vander Lugt[5] & Jerry Vockley[1,2]

Supplementary Data

Case Description

The patient is a 5-year-old Amish boy with a history of failed newborn hearing screen in the right ear and 6 prior otitis media who presented at 10 months of age with sepsis and pneumonia. He was born at term to a G0P0 mother and his birthweight was 2.8 kg (12th percentile). His immunizations were up to date until 6 months of age. His parents were second cousins, and there was a history of distant relatives with "dwarfism" but no history of neutropenia, immuno-deficiency, or recurrent infections. Blood culture on presentation grew *Haemophilus influenza* and *Pseudomonas aeruginosa* and respiratory viral testing was positive for human metapneumo-virus. He was noted to have neutropenia and lymphopenia, as well as low IgG, elevated IgA, and normal IgM. Vaccine response was intact to diphtheria and tetanus, but absent to pneumococcus. Lymphocyte proliferation was normal to pokeweed mitogen (PWM), but decreased to phyto-hemagglutinin (PHA). His parents reported that his newborn screen was normal, although he was later confirmed to have absent TREC on the newborn screen. T cell receptor (TCR) Vβ spectrotyping showed a normal TCR repertoire (See table S1 for detailed laboratory evaluation). The patient's physical exam at presentation was remarkable for a weight of 5.6 kg ($<1^{st}$ percentile), respiratory distress, and hepatomegaly.

The patient presented again at 13 months of age with culture-negative septic shock and a rhinovirus pneumonia. He was again noted to be neutropenic and lymphopenic. A bone marrow aspirate and biopsy showed maturation through the promyelocyte/myelocyte stage with rare segmented neutrophils, and he was started on G-CSF for neutropenia. He continued to have CD4 lymphopenia with decreased numbers of B and NK cells but normal numbers of

CD8 cells. Genetic evaluation revealed duplication of chromosome 16p11.2, which has been observed in autistic patients but was deemed unlikely to have clinical relevance to the patient's disease presentation. Regions of homozygosity in chromosomes 1, 2, and 10 were also identified with possible genes of interest in these regions including *LCK*, *IFNLR1*, *FCN3*, *AK2* and *DPP4*. Further genetic testing was performed for the founder mutations in genes associated with immune deficiency disorders that are commonly seen in the Amish populations, all were normal. Sequencing of *ELANE* gene was also normal (**Table S1**).

The neutropenia was initially managed with daily G-CSF injections and maintained an ANC of 800-6000 at G-CSF dosing of approximately 2-3 mcg/kg. At 20 months of age, he developed Sweet syndrome, requiring discontinuation of G-CSF. Off G-CSF, he maintained an ANC between 900-1900 cells/μL, but would decrease to 300-400 cells/μL with viral infections. He was started on IVIG for hypogammaglobulinemia. A CT scan showed bronchiectasis likely due to chronic pulmonary infections. At 3 years of age, he developed primary CMV viremia which had poor response to ganciclovir and foscarnet. Repeat testing at that time showed normal numbers of $CD3^{+}$ and $CD8^{+}$ T cells, but continued $CD4^{+}$ Lymphopenia and low B cells. Repeat mitogen stimulation was decreased to PHA, Concanavalin A (ConA), and Pokeweed mitogen (PWM). Blood for whole exome sequencing was drawn. Due to a lack of suitable unrelated and umbilical cord blood donors, he underwent a reduced-intensity 7/8 HLA-matched related donor bone (maternal) marrow transplant at 3 years of age because of his persistent CMV viremia and neutropenia. His transplant course was complicated by CMV and adenoviremia, grade II acute skin GVHD and extensive chronic skin GVHD treated with systemic and topical corticosteroids, tacrolimus, methotrexate, and basiliximab. On this therapy, he developed a multifocal non-mucor zygomycete infection of the lungs, kidney, and pancreas requiring surgical resection and therapy

with liposomal amphotericin and IV posaconazole. In addition, although he engrafted with full donor chimerism and maintained a normal neutrophil count in the absence of G-CSF, he eventually developed neutropenia and complete recipient chimerism in the myeloid lineage by 6 months post-transplant. His CD3 chimerism remained fully donor throughout his post-transplant course. Because of the zygomycete infection and neutropenia, he was re-started on G-CSF which was complicated by splenomegaly with secondary anemia and thrombocytopenia requiring transfusion support and improved with cessation of G-CSF. The patient was evaluated by otolaryngology at 4 years of age for abnormal speech and found to have bilateral moderate to severe sensorineural hearing loss. Post-transplant, he developed hypopigmentation/ depigmentation of the hair in his eyebrows and the anterior part of the scalp with normal pigmentation of the remainder of his hair. Because of his fungal infection and persistent neutropenia, 2 years after his first transplant he underwent a CD3/CD19 depleted peripheral blood transplant from the same donor using busulfan as conditioning. This transplant was complicated by engraftment syndrome and fatal severe veno-occlusive disease of the liver.

Supplementary Table S1. Laboratory evaluation of the patient

**Hematology**	**Birth**	**10 mo**	**11 mo**	**13 mo**	**2 yrs**	**3 yrs (pre-BMT)**	**Reference**
WBC count (cells*10^9/L)		1.1	3.0	0.6	3.1	4.6	6-17.5
ANC (cells*10^9/L)		0.44	1.0	0.07	1.8	0.60	1-8.5
ALC (cells*10^9/L)		0.20	1.7	0.47	1.0	3.70	4-13.5
**Lymphocyte subsets**							
CD 3^+ T cells/μL		143	966			3,066	2,400-3,300
$CD4^+$ T cells/μL		15	163		289	245	1,600-2,200
$CD4^+CD45RA^+CD62L^+$ Tcells (%)						15	
$CD8^+$ T cells/μL		118	708			2,342	820-1,600
$CD19^+$ B cells/μL		15	644			146	1,000-1,600
$CD16/56^+$ NK cells/μL		3	116			153	270-1,100
**Lymphocyte proliferation**							
PWM (CD45)			5.5%	2.5%			>4.5%
PWM (CD3)			6.2%	2.9%			>3.5%
PWM (CD19)			6.7%	1.6%			>3.9%
PHA (CD45)			25.2%	7.9%			>49.9%
PHA (CD3)			28.4%	12.4%			>58.5%
Viability			82.6%	66%			>75%
**TREC**							
TREC (newborn screen)	<7 copies						
TREC (copies/10^6 $CD3^+$ cells)				1980			>4169
**Immunoglobulin levels**							
IgM (mg/dL)		55	28	31	12	15	11-99
IgG (mg/dL)		211	255	248	660	770	330-1,133
IgA (mg/dL)		281	688	671	323	413	15-97
**Vaccine Response**							
Anti-tetanus antibody (IU/ml)		0.40		<0.10			>0.15
Anti-diphtheria antibody (IU/ml)		0.03		0.02			>0.01
Pneumococcal antibodies		Absent response to 13/23 serotypes					
**Red Blood Cell Enzymes**							
ADA (nmol/h/mg)		63.5					63+/-41.4
PNP (nmol/h/mg)		1731					1336+/-441
**Additional Testing**							
SAP expression		Normal					
XIAP expression		Normal					
MHC Class I and II expression		Normal					
**Targeted Genetic Testing**							
RAG1 (c.2974A>G;p.Lys992Glu)			No mutation				
ADA (c.646G>A;p.Gly216Arg)			No mutation				
RMRP (c.70A>G)			No mutation				
ITCH (c.394dupA)			No mutation				
ELANE (complete gene sequence)			No mutation				

Supplementary Table S2. List of Oligonucleotides

	**Oligonucleotide sequence**	**Sequence 5´… 3´**	**Band size**
AK2 gene (genomic DNA sequence)	*AK2* forward	TGATTCACCCCAAGAGTGGC	1668 bp
	AK2 reverse	GCTGGTAAAGCAACCTAGCCT	
AK2 gene (RT-PCR)	*AK2* forward	GGCTTTGGAGAAGGCTGCTA	132 bp
	AK2 reverse	CTGCAAGCCTACCACACTCA	
GAPDH gene (RT-PCR)	*GAPDH* forward	TGGCATTGCCCTCAACGACC	102 bp
	GAPDH reverse	TACTCCTTGGAGGCCATGTGG	

Supplementary Table S3. List of Antibodies

Anti-AK2 (western blot)	Purified rabbit anti human AK2 monoclonal antibody
	Catalog number: ab157206
	Abcam, Cambridge, MA, USA
Anti-β-actin	Purified mouse anti human β-actin monoclonal antibody
	Catalog number: A1978
	Sigma-Aldrich, St. Louis, MO, USA
Goat anti-rabbit IgG (H+L)-HRP-conjugate	Catalog number: 170-6515
	BioRad, Munich, Germany
Goat anti-mouse IgG-HRP-Conjugate	Catalog number: sc-2005
	Santa Cruz, Santa Cruz, CA, USA
Anti-AK2 (immunofluorescence staining)	Purified rabbit anti human AK2 polyclonal antibody
	Catalog number: ab37594
	Abcam, Cambridge, MA, USA
Anti-MTCO1 (immunofluorescence staining)	Purified mouse anti-MTCO1 monoclonal antibody
	Catalog number: ab14705
	Abcam, Cambridge, MA, USA
Donkey anti-rabbit IgG (H+L) secondary antibody Alexa Fluor 488	Catalog number: A21206
	ThermoFisher Scientific, Waltham, MA, USA
Donkey anti-mouse IgG secondary antibody Alexa Fluor 647	Catalog number: A31571
	ThermoFisher, Waltham, MA, USA

Supplementary Figure S1: Western blotting with Anti-AK2 antibody on CT and PT with different protein load from whole cell (fibroblasts) lysates (10, 25 and 50 µg protein).

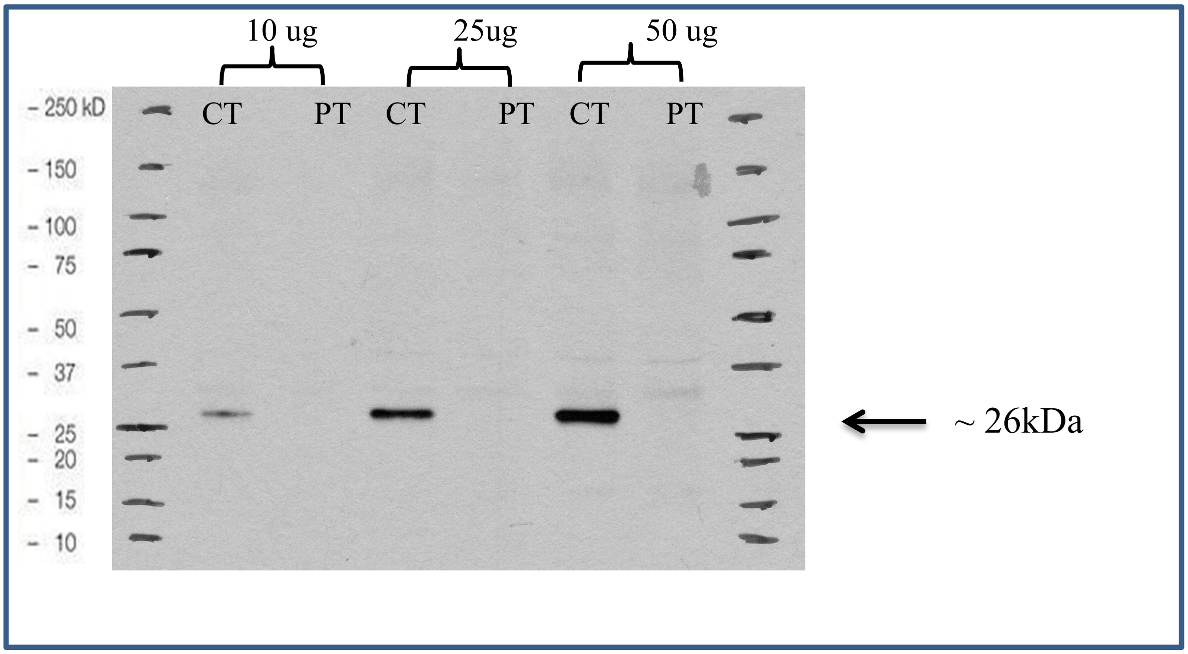

Supplementary Figure S2: Western blotting with Anti-β-actin antibody on CT and PT with different protein load from whole cell (fibroblasts) lysates (10, 25 and 50 µg protein).

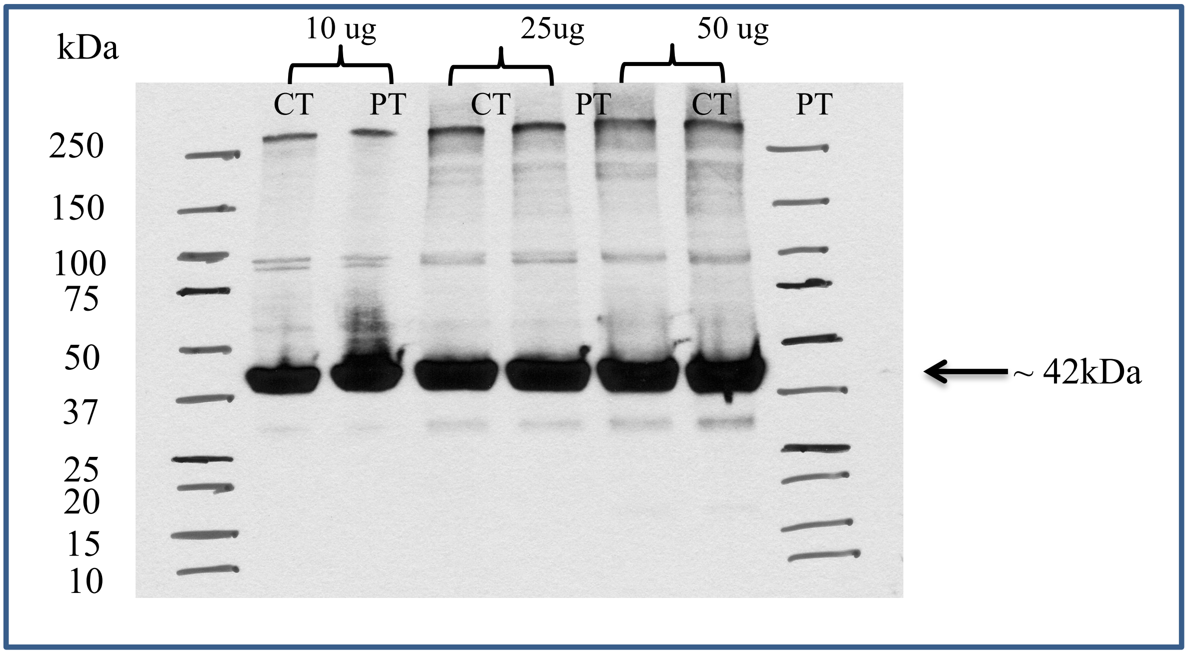

Supplementary Figure S3: RT-PCR with AK2 and GAPDH primers on CT, PT and negative control (H_2O) samples.

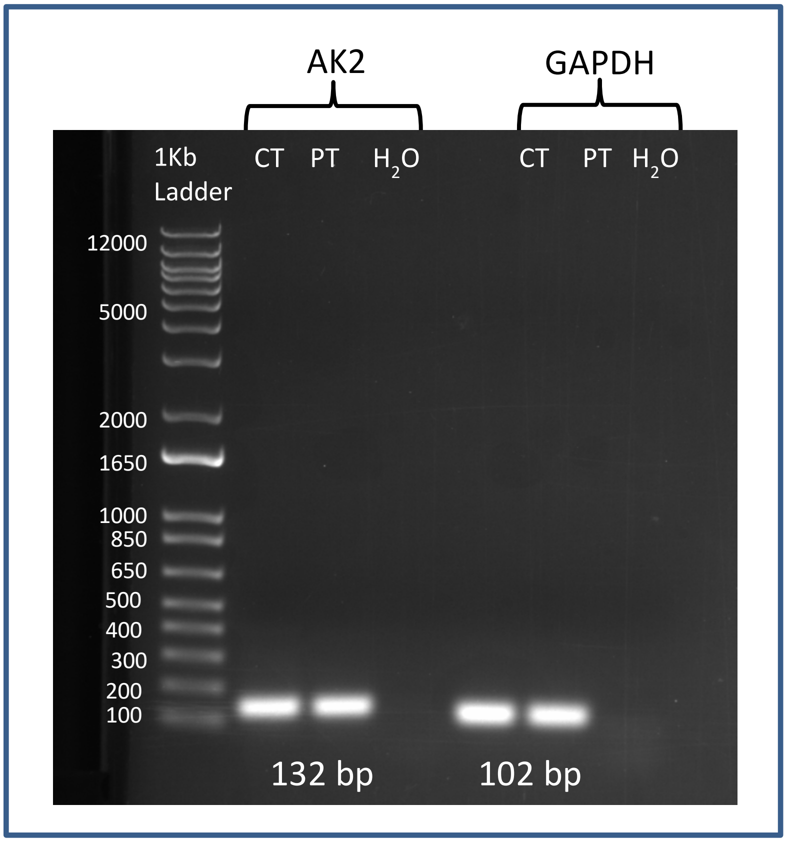

Supplement: Supplementary file 1 — Supplementary Materials [file 41598_2019_51922_MOESM1_ESM.pdf]
